# Upregulated Solute Carrier SLC39A1 Promotes Gastric Cancer Proliferation and Indicates Unfavorable Prognosis

**DOI:** 10.1155/2022/1256021

**Published:** 2022-11-04

**Authors:** Dan Yu, Yong Chen, Ming Luo, Yanjin Peng, Shengen Yi

**Affiliations:** Department of General Surgery, The Second Xiangya Hospital of Central South University, Changsha 410011, China

## Abstract

**Backgrounds:**

Solute carrier 39A1 (SLC39A1) is an indirect zinc transporter which showed diverse tumor-related functions in different malignancies. Here, we aimed to investigate its expression and role in gastric adenocarcinoma.

**Methods:**

A retrospective gastric adenocarcinoma cohort (*n* = 154) was collected from our hospital to test their tissue expression of SLC39A1 through immunohistochemical staining method. After SLC39A1 overexpression or knockdown, proliferation and invasion assays were conducted for proliferation and invasion estimation, respectively. Xenograft in nude mice was used as the in vivo strategy to validate in vitro findings.

**Results:**

Compared with adjacent stomach tissues, gastric adenocarcinoma tissues showed significantly higher SLC39A1 on both mRNA and protein levels. Higher SLC39A1 was observed in patients with larger tumor size (*P*=0.003) and advanced tumor stages (*P* < 0.001). Univariate (*P*=0.001) and multivariate analyses (*P*=0.035) confirmed the independent prognostic significance of SLC39A1 on gastric adenocarcinoma outcomes. The median survival time was 22.0 months in patients with high-SLC39A1 expression, while up to 57.0 months in those with low-SLC39A1 (*P*=0.001). *In vitro* and *in vivo* assays demonstrated that overexpressing SLC39A1 could promote gastric cancer growth and invasion, while silencing SLC39A1 led to opposite effects.

**Conclusions:**

Aberrant high-SLC39A1 expression can serve as an independent unfavorable prognostic factor for gastric adenocarcinoma. High SLC39A1 is critical for a more aggressive tumor phenotype by promoting cell proliferation and invasion. Therefore, targeting SLC39A1 may provide novel therapeutic insights.

## 1. Introduction

Gastric cancer is the second most common malignancy of the digestive system [1]. Despite screening and management of gastric cancer have been improved in the past decades, it ranks the third leading cause of tumor-related death worldwide [2]. Surgical intervention is the most recommended therapy for early-stage gastric cancers. However, a large amount of patients were diagnosed at local advanced stage or with distant metastasis; thus, losing the best opportunity for radical surgery. Although remarkable achievements had been made regarding chemotherapies and targeted therapies, the overall prognosis of gastric cancer remains far from satisfied due to high occurrence of disease relapse and metastasis. In general, the 5-year overall survival of gastric cancer is no more than 30%, and those with advanced stages were even lower [3]. More biomarkers for gastric cancer diagnosis, prognostic prediction, and therapeutic development are in urgent need.

The solute carrier (SLC) is a large protein family that contains more than 400 members, most of which localize on the plasma membrane and assist solute transportation [4]. Among them, SLC39A subfamily is specific for zinc transportation, thus also named zinc transporter (ZIP) family [5]. SLC39A1 is the first identified SLC39A family member, which scientists suggest may have indirect zinc uptake function as indicated by SLC39A1-knockout mice studies [6]. Another evidence is that SLC39A1 has varied subcellular localizations in addition to cellular membrane, such as vesicular and cytoplasm [7]. Nevertheless, SLC39A1 indeed regulates the zinc homoeostasis in human gut epithelial cells since its overexpression resulted in enhanced zinc accumulation in Caco2 cells. Dysregulated expression of SLC39A1 was reported to be correlated with multiple disorders, including short stature [8], chronic inflammation [9], and chronic obstructive pulmonary disease (COPD) [10]. Considering that exogenous zinc has significant effect on cell viability and apoptosis [11, 12], its potential role in carcinogenesis is attracting more and more attention. Recently, hyper-expression of SLC39A1 has been reported to participate in the malignant progression of hepatocellular carcinoma and glioma [13, 14]. However, its role in gastric cancer remains unknown.

Here, we first compared the different expression of SLC39A1 between gastric adenocarcinoma tissues and nontumorous stomach tissues. Its correlations with tumor characteristics and disease prognosis were also evaluated using a retrospective cohort from our hospital. The tumor-related role of SLC39A1 in gastric cancer was finally proven through *in vitro* and *in vivo* experiments.

## 2. Methods

### 2.1. Online Database

We used the GEPIA resource to analyze the differential mRNA level of SLC39A1 between gastric adenocarcinomas and adjacent normal stomach tissues. Information of gastric adenocarcinoma patients and healthy individuals was retrieved from the Cancer Genome Atlas (TCGA) and genotype-tissue expression (GTEx), respectively.

### 2.2. Patients and Samples

Primary gastric adenocarcinoma tissues were collected by surgical resection from our department. All tumor diagnoses were based on pathological test according to World Health Organization criteria. All the enrolled cases (*n* = 154) were retrieved with intact clinicopathological information including patients' age, sex, tumor localization, tumor diameter, tumor differentiation, *T* stage, and lymph node metastasis. Overall survival (OS) was defined as the interval between the dates of surgery and death or the last date of follow-up.

### 2.3. Immunohistochemistry (IHC) Stain

IHC staining was conducted as previously described [15]. The staining percentage was scored as 0, 1, 2, and 3 for the positive staining percentage of 0%, 1%-25%, 26%-50%, and 51-100%, respectively. The staining intensity was scored as 0 (negative), 1 (weak), 2 (moderate), or 3 (strong). A final score ranging 0-9 was calculated by multiplying the staining percentage score with the intensity score. Based on the median score, all patients were equally divided into the low-SLC39A1 group (*n* = 77) or high-SLC39A1 group (*n* = 77).

### 2.4. Cell Culture and Infection

MKN28 and MKN45 cell lines were purchased from ATCC. Both cell lines were grown in DMEM containing 10% fetal bovine serum (FBS, Gibco), 100 U/ml penicillin, and 100 mg/ml streptomycin at 37°C in an incubator with 5% CO_2_. Lentivirus infection strategy was used to obtain SLC39A1 knockdown and SLC39A1 overexpression cell lines. Briefly, the specific shRNA and overexpressing plasmids were transfected according to the manufacture's procedure. Stable transfected cell lines were validated by puromycin (2 mg/mL) selection.

### 2.5. Western Blotting

Total protein from cultured cells was extracted with RIPA lysis buffer containing protease and phosphatase inhibitors. Extracted proteins were separated with 10% SDS-PAGE and transferred them onto PVDF membranes. The membranes were then blocked with nonfat milk and incubated with primary antibodies in a cold room overnight. On the next day, membranes were incubated with a secondary antibody and visualized using enhanced chemiluminescence (ECL) reagent [16].

### 2.6. Cell Proliferation

Stable cells were seeded in 96-well plates at 3 × 10^3^ cells/well and cultured for 24, 48, 72, or 96 hours. Cell proliferation was then estimated using an MTT method according to the standard procedure.

### 2.7. Cell Migration and Invasion

Cell migration and invasion were assessed by transwell assay. Briefly, 2 × 10^4^ cells in serum-free DMEM were seeded on a membrane (8.0 *µ*m pores) in a 24-well plate, while DMEM containing 10% FBS was added to the lower chamber. After 48 hours, cells that had reached the underside of the chamber were stained with Giemsa and counted under a light microscope. For the invasion assay, 10 *µ*L of Matrigel (BD Biosciences) was preadded to each well 6 hours before cell seeding.

### 2.8. Mice Xenografts

Gastric adenocarcinoma cells were injected subcutaneously into the flanks of 6-week-old BALBc nu/nu mice. All mice were cultivated with free food and water in a specific pathogen-free environment. Tumor's largest (a) and smallest (b) diameters were measured every five days.

Tumor volume was calculated as follows: volume = (*a* × *b*^2^)/2. After one month, mice were sacrificed and tumors were excised for picturing and weighting.

### 2.9. Statistics

Clinical data were assessed using SPSS 22.0 Software with Chi-square test, Kaplan–Meier log-rank test, and Cox proportional hazard regression test. All experimental assays were independently conducted at least three times and analyzed with GraphPad Prism 7.0 software. Student's *t*-test or one-way analysis of variance (ANOVA) was performed to determine significance. Data were shown as mean ± SD. A *P* value < 0.05 was considered significant.

### 2.10. Ethical Approval

Ethical approval for the use of human specimen and mice experiments was obtained from the Research Ethics Committee of the Second Xiangya Hospital of Central South University. The trial registration number was ChiCTR202132254. Informed consent of each patient was obtained.

## 3. Results

### 3.1. The mRNA Level and Prognostic Role of SLC39A1 in Online Dataset

**Figure 1:**
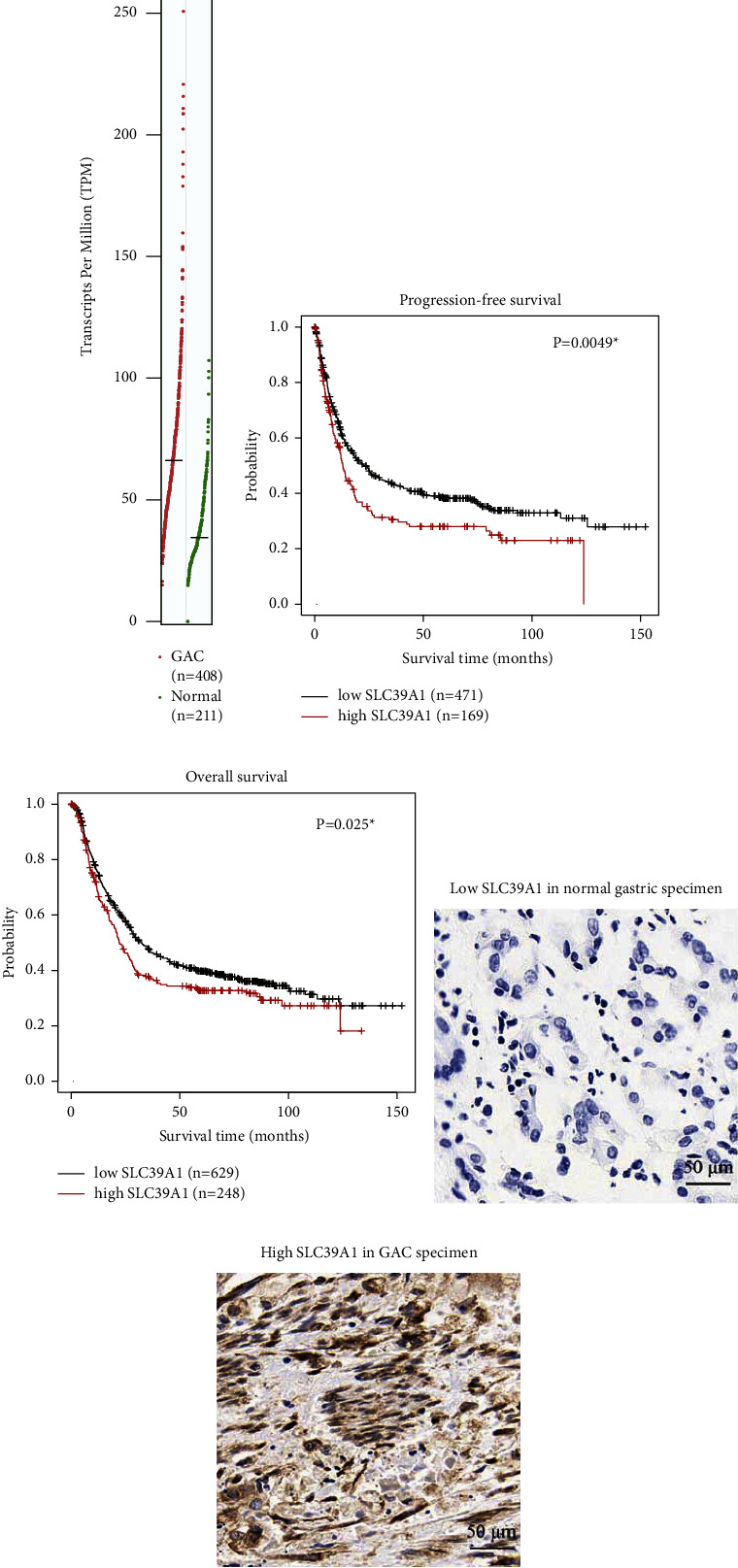
Upregulated expression of SLC39A1 in gastric adenocarcinoma. (a) The mRNA level of SLC39A1 was extracted from TCGA and GTEx datasets and plotted by GEPIA server (https://gepia.cancer-pku.cn/index.html) according to transcripts per million (TPM). The data showed that SLC39A1 mRNA level was higher in gastric adenocarcinoma (GAC) tissues than that in normal stomach tissues. (b) Kaplan–Meier progression-free survival and overall survival (c) curves were plotted using the K-M plot server (https://kmplot.com/analysis/) according to the mRNA level of SLC39A1, which implied that higher SLC39A1 helped predict worse progression-free survival and overall survival. (d) Representative IHC image showed low expression of SLC39A1 protein in adjacent stomach tissues. (e) Representative IHC image exhibited high expression of SLC39A1 protein in gastric adenocarcinoma tissues. *∗* indicates *P* < 0.05. Magnification: 400X.

The mRNA level of SLC39A1 was retrieved from TCGA dataset and GTEx dataset as described in the Methods section. According to the transcripts per million (TPM), gastric cancer tissues possessed significantly higher SLC39A1-mRNA level than normal stomach tissues (*P* < 0.001, Figure 1(a)). In addition, Kaplan–Meier survival curves indicated that higher SLC39A1-mRNA was correlated with unfavorable progression-free survival and overall survival. The median progression-free survival time of the low-SLC39A1-mRNA group was 23.2 months, while it was decreased to 13.0 months in the high-SLC39A1-mRNA group (Figure 1(b), *P*=0.005). Similarly, the median overall survival time of the low-SLC39A1-mRNA group was 32.1 months, while it was decreased to 22.3 months in the high-SLC39A1-mRNA group (Figure 1(c), *P*=0.025).

### 3.2. Protein Expression and Clinical Relevance of SLC39A1 in Gastric Adenocarcinoma

Besides analyzing the mRNA level of SLC39A1 in the samples from online database, we enrolled an independent cohort of gastric adenocarcinoma patients from our hospital (*n* = 154). By immunohistological staining, we found that SLC39A1 was lowly expressed in adjacent stomach tissues (Figure 1(d)), while highly stained in the gastric adenocarcinoma specimen (Figure 1(e)). As described above, all enrolled patients were equally subgrouped as the low-SLC39A1 group (*n* = 77) or high-SLC39A1 group (*n* = 77) according to the IHC data.  Table 1 exhibits the basic characteristics of all cases. Chi-square test indicated a positive correlation between SLC39A1 level and patients' *T* stage (*P* < 0.001). Meanwhile, patients with positive lymph node metastasis were more prevalent to express higher SLC39A1 level (*P*=0.018). However, no statistical significance was identified on the correlation between SLC39A1 and other variables.

### 3.3. SLC39A1 is an Independent Prognostic Factor in Gastric Adenocarcinoma

**Figure 2:**
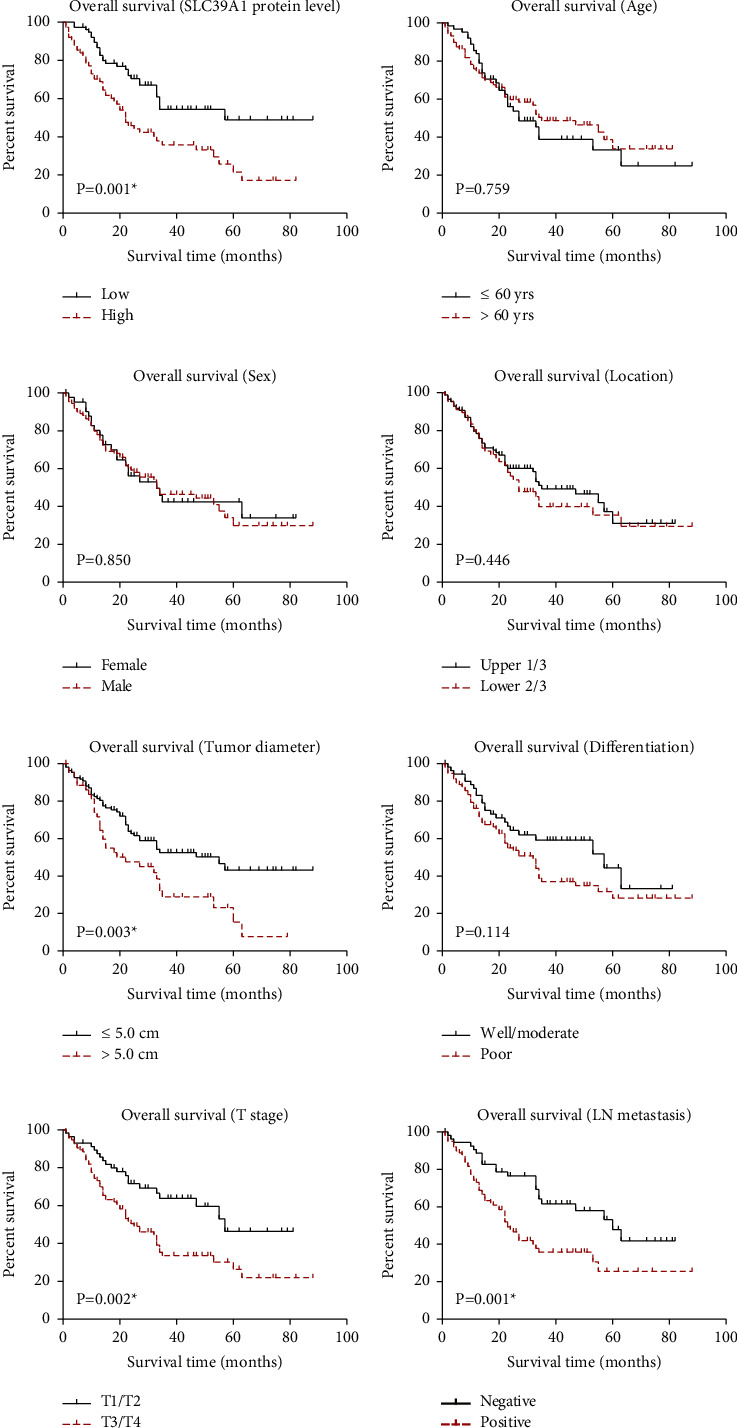
Overall survival curves of gastric adenocarcinoma patients in our retrospective cohort. The survival curves were plotted using Kaplan–Meier method and compared using log-rank test based on SLC39A1 level (a), patients' age (b), sex (c), tumor localization (d), tumor diameter (e), tumor differentiation (f), T stage (g), and lymph node metastasis (h). *∗* indicates *P* < 0.05.

Considering the clinical relevance of SLC39A1 in gastric adenocarcinoma, we next conducted survival analyses of our retrospective cohort using Kaplan–Meier method (Figure 2, Table 2). Consistent with the results from TCGA dataset, higher SLC39A1 protein level was significantly correlated with worse overall survival in our cohort (Figure 2(a), *P*=0.001). The 5-year overall survival rate of the low-SLC39A1 group was 49.0%, while it was only 21.2% in the high-SLC39A1 group (Table 2). The median overall survival time of the low-SLC39A1 group was 57.0 months, while it decreased to 22.0 months in the high-SLC39A1 group. However, patients' age, sex, or tumor location showed no significant correlation with clinical outcomes (Figures 2(b)–2(d)). As a conventional prognostic factor, tumor size also affected the overall survival of gastric cancer (*P*=0.003, Figure 2(e)). Despite patients with poor histological differentiation grade (median survival time 32.0 months) showed worse overall survival compared to those with well or moderate differentiation grade (median survival time 57.0 months), our data did not identified its statistical significance (Figure 2(f), *P*=0.114). As expected, patients' *T* stage (*P*=0.002) and lymph node metastasis (*P*=0.001) both exerted remarkable effects on the overall survival (Figures 2(g) and 2(h)).

Cox proportional hazard regression analysis was then performed to identify independent risk factors using all the significant variables mentioned above (Table 3). Despite tumor diameter and *T* stage lost their significance, lymph node metastasis was identified to be an independent risk factor (hazard ratio 1.815, 95% confident interval 1.067-3.087, *P*=0.028). Importantly, SLC39A1 was also validated as a novel independent prognostic biomarker for gastric adenocarcinoma (hazard ratio 1.732, 95% confident interval 1.041-2.881, *P*=0.035).

### 3.4. SLC39A1 Plays Pro-oncogenic Effects in Gastric Adenocarcinoma Cell Lines

**Figure 3:**
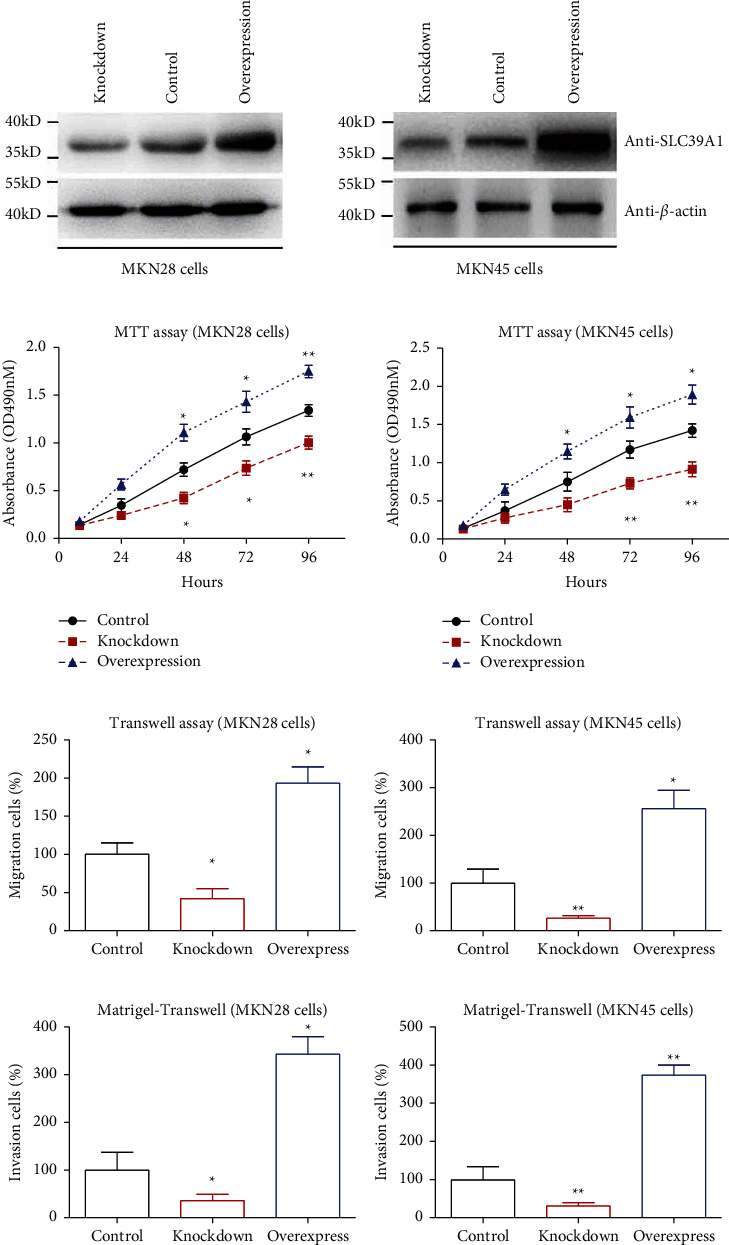
SLC39A1 showed oncogenic effects in gastric adenocarcinoma cells. (a) Transfection efficiencies of SLC39A1 overexpression, SLC39A1-knockdown, and pLKO.1 vector control cells were tested via Western blotting in MKN28 and MKN45 cells, respectively. (b) MTT experiments were conducted to test the effects of silencing or overexpressing on gastric adenocarcinoma cell proliferation. (c) Migration capacities of gastric adenocarcinoma cell lines were tested by Transwell assays. (d) Invasion capacities of gastric adenocarcinoma cell lines were evaluated by Matrigel-Transwell assays. Data was presented as Mean ± SD from three independent repeats. *∗* indicates *P* < 0.05 by Student's *t*-test compared with vector control cells.

Since clinical data analyses emphasized the potential involvement of SLC39A1 in gastric cancer, we were engaged to dig into its detailed effects using *in vitro* cell lines, including MKN28 cells and MKN45 cells. Western blotting was first conducted to test the efficiencies of knockdown and overexpression (Figure 3(a)). Afterwards, MTT assays demonstrated that overexpressing SLC39A1 promoted cell proliferation in both cell lines, while silencing SLC39A1 inhibited this process (Figure 3(b)). Additionally, the migration and invasion capacities were impaired by SLC39A1 knockdown, while they were enhanced by SLC39A1 overexpression (Figures 3(c) and 3(d)). In other words, transwell experiments implied that SLC39A1 participates in tumor metastasis, which was consistent with clinical finding that SLC39A1 was positively correlated with lymph node metastasis.

### 3.5. SLC39A1 Promotes Gastric Adenocarcinoma Growth *in Vivo*

**Figure 4:**
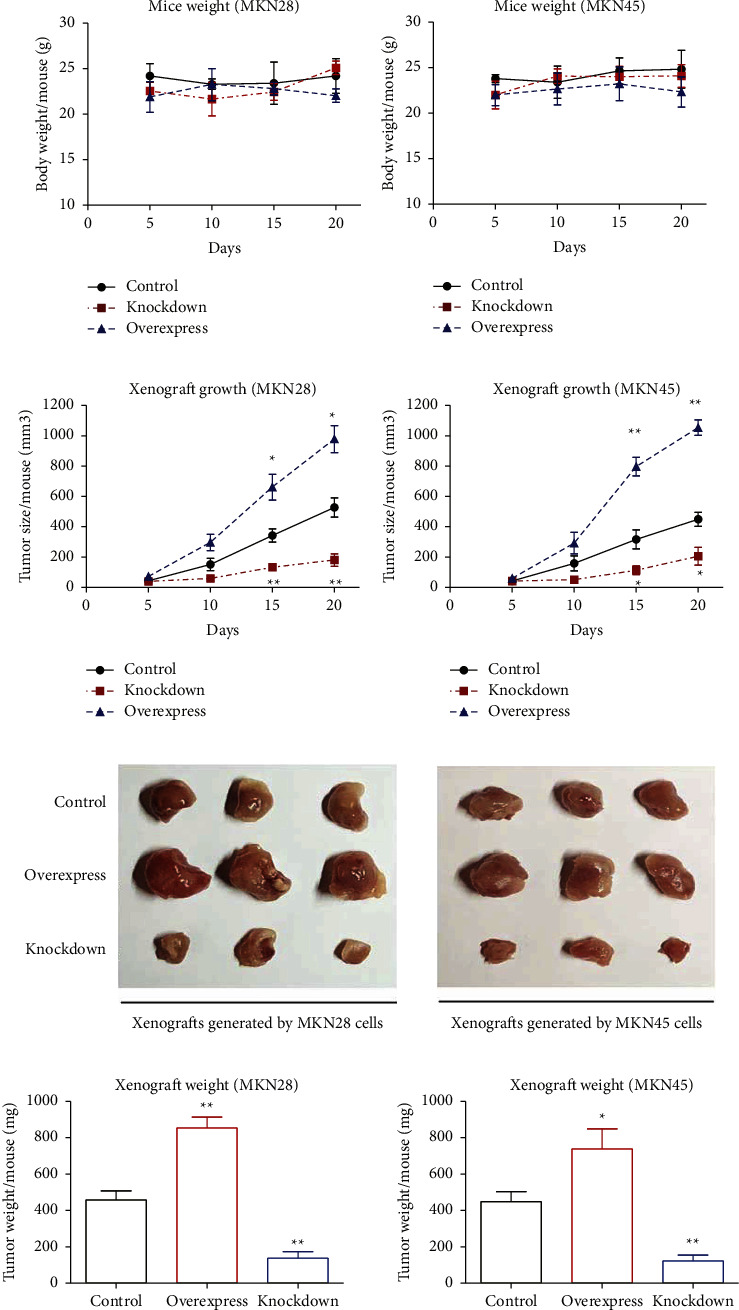
SLC39A1 promoted gastric adenocarcinoma growth *in vivo*. Transfected MKN28 or MKN45 cells were subcutaneously injected into nude mice and then the mice weight (a) and xenograft volumes (b) were monitored every five days. Three weeks after cell injection, all mice were sacrificed to isolate the xenografts for picturing (c) and weighting (d). Data were presented as mean ± SD from three independent repeats. *∗* indicates *P* < 0.05 by Student's *t*-test compared with vector control group.

To further confirm the tumor-related role of SLC39A1 in gastric cancer, we finally conducted *in vivo* experiments using nude mice models. After subcutaneous injection of tumor cells, mice were weighted every five days to monitor their nutrition status, which showed no statistical difference (Figure 4(a)). In contrast, *in vivo* tumor growth was significantly suppressed in the SLC39A1-knockdown group, while it was accelerated in the SLC39A1-overexpression group (Figure 4(b)). Consistently, after excision, the tumor volume was significantly smaller in the SLC39A1-knockdown group and larger in the SLC39A1-overexpression group (Figure 4(c)). The same prevalence was observed regarding isolated tumor weight (Figure 4(d)).

## 4. Discussion

Gastric adenocarcinoma is characterized with unsatisfied prognosis due to rapid tumor progression and metastasis. Identifying novel biomarkers and elucidating systematic functional mechanisms are still in urgent demand. Here, we demonstrated that SLC39A1 was upregulated in gastric cancer tissues, and its hyper-expression was correlated with unfavorable prognosis. Consistent with its dysregulation in gastric cancer, a previous study reported that SLC39A1 was significantly upregulated in breast cancer tissues compared with normal breast tissues [17]. However, in silico analyses did not find its correlation with breast cancer prognosis [17, 18]. Recently, Wang's study revealed that SLC39A1 was highly expressed in gliomas and negatively correlated with glioma outcome [14], highlighting its involvement in different malignancies. As for the detailed tumor-related functions, our *in vitro* and *in vivo* data proved that SLC39A1 overexpression can promote gastric adenocarcinoma proliferation, while SLC39A1 knockdown suppressed tumor growth. Consistent with our findings, Wang et al. demonstrated that SLC39A1 promoted proliferation of glioma cells and inhibited their apoptosis [14].

In contrast with its oncogenic role in gastric cancer and glioma, SLC39A1 has been shown to exert tumor suppression effects in prostate cancer [19–21]. The distinct role of SLC39A1 in prostate cancer may be partially explained by the fact that the prostate gland contains extremely high zinc levels and evolves with unique functions [22]. The high zinc level may have specific crosstalk with SLC39A1, therefore shows specific tumor-related functions. Indeed, enhanced zinc uptake and accumulation may inhibit prostate cancer progression [23]. Besides prostate cancer, the function of SLC39A1 seems controversial in hepatocellular carcinoma (HCC). On one hand, Zhang et al. reported that SLC39A1 was decreased in early-stage HCC and low-SLC39A1 predicted unfavorable prognosis [24]. On the other hand, Ma's group suggested that SLC39A1 was overexpressed in HCC tissues, and they showed a significant correlation between high-SLC39A1 and worse HCC prognosis. Their bioinformatics analyses and experimental data found that SLC39A1 knockdown suppressed the expression of cyclin D1, MMP2, Wnt3A, and *β*-catenin and subsequently inhibited HCC progression. Moreover, overexpressing SLC39A1 may affect tumor immunity in HCC, as indicated by the enhanced infiltration of Th2 cells and impaired infiltration of cytotoxic cells [13]. Of note, involvement of SLC39A1 in modulating infiltration of immune cells was also suggested in glioma microenvironment [14]. Therefore, further studies will be invaluable to investigate whether SLC39A1 can regulate immune microenvironment of gastric adenocarcinomas.

Despite our data did not find any significant expression correlation with patients' age in gastric tumor tissues, SLC39A1 was reported to show age-related alterations in human brain tissues [25]. According to Olesen Data, expression of SLC39A1 was significantly increased in elder individuals, while it decreased in cases with higher body mass index (BMI). However, whether this expression alteration is applicable in human stomach tissues remains unknown. Interestingly, Desouki and his colleagues reported that SLC39A1 showed lower expression in gastric mucinous carcinoma than that of gastric adenocarcinoma [26]. Although their data only enrolled 31 gastric mucinous carcinomas and 4 gastric adenocarcinomas, the conclusion may be convincible since a similar difference was observed in other tumor types including ovarian cancer and colon cancer. In the current study, the cohort we selected was gastric adenocarcinoma patients, and cell lines were also established from human gastric adenocarcinoma; further comparing and understanding its distinct expression in different histological types would be necessary.

## 5. Conclusions

SLC39A1 is upregulated in gastric adenocarcinoma and plays oncogenic functions by promoting tumor growth and metastasis. Targeting SLC39A1 may not only help predict patients' prognosis but also facilitate therapeutic development.

## Figures and Tables

**Table 1:** Correlations between SLC39A1 protein level with patients' characteristics.

Characteristic	Case (*n* = 154)	SLC39A1 protein level		*P*-value
		Low (*n* = 77)	High (*n* = 77)	
Age				
≤60 yrs	64	30	34	0.513
>60 yrs	90	47	43	

Sex				
Female	43	17	26	0.106
Male	111	60	51	

Localization				
Upper 1/3	85	46	39	0.257
Lower 2/3	69	31	38	

Tumor diameter				
≤5.0 cm	109	63	46	0.003^*∗*^
>5.0 cm	45	14	31	

Differentiation				
Well/moderate	56	29	27	0.738
Poor	98	48	50	

T stage				
T1/T2	58	43	15	<0.001^*∗*^
T3/T4	96	34	62	

LN metastasis				
Negative	54	34	20	0.018^*∗*^
Positive	100	43	57	

**Table 2:** Kaplan–Meier overall survival (OS) analyses.

Characteristic	Case (*n* = 154)	Median OS (months)	5-year OS (%)	*P*-value
Age				
≤60 yrs	64	27.0	33.3	0.759
>60 yrs	90	34.0	33.5	

Sex				
Female	43	33.0	42.4	0.850
Male	111	33.0	29.7	

Localization				
Upper 1/3	85	35.0	31.1	0.446
Lower 2/3	69	27.0	34.9	

Tumor diameter				
≤5.0 cm	109	55.0	43.1	0.003^*∗*^
>5.0 cm	45	19.0	22.6	

Differentiation				
Well/moderate	56	57.0	43.6	0.114
Poor	98	32.0	28.2	

T stage				
T1/T2	58	57.0	46.4	0.002^*∗*^
T3/T4	96	24.0	26.1	

LN metastasis				
Negative	54	60.0	47.9	0.001^*∗*^
Positive	100	23.0	25.3	

SLC39A1 protein level				
Low	77	57.0	49.0	0.001^*∗*^
High	77	22.0	21.2	

**Table 3:** Cox hazard regression multivariate analysis.

Characteristic	Hazard ratio	95% CI	*P*-value
Tumor diameter			
≤5.0 cm	Reference		
>5.0 cm	1.496	0.915-2.446	0.108

T stage			
T1/T2	Reference		
T3/T4	1.135	0.608-2.118	0.691

LN metastasis			
Negative	Reference		
Positive	1.815	1.067-3.087	0.028^*∗*^

SLC39A1 protein level			
Low	Reference		
High	1.732	1.041-2.881	0.035^*∗*^

## Data Availability

Data will be made available upon reasonable request to the corresponding author.
